# Diabetes knowledge levels among patients in Mhlontlo, South Africa: A quantitative study

**DOI:** 10.4102/safp.v67i1.6183

**Published:** 2025-12-19

**Authors:** Zimkhitha Diniso, Nongiwe L. Mhlanga, Monwabisi Faleni

**Affiliations:** 1 Department of Public Health, Faculty of Medicine and Health Sciences, Walter Sisulu University, Mthatha, South Africa

**Keywords:** diabetes, knowledge, Mhlontlo, Eastern Cape, diabetes self-management

## Abstract

**Background:**

There is a high prevalence of diabetes in South Africa and a related increase in poor treatment outcomes among people with diabetes. Poor glycaemic control is often associated with a lack of knowledge of self-management. The study aimed to assess diabetes knowledge levels among patients in the Mhlontlo Municipality in the OR Tambo District of the Eastern Cape province in South Africa.

**Methods:**

The study used a quantitative descriptive cross-sectional design. A convenience sample was taken of patients ≥ 18 years of age with Type 2 diabetes at a Community Health Centre and a district hospital. Data were analysed using SPSS 29, with descriptive statistics and chi-square tests applied.

**Results:**

A total of 172 respondents were surveyed. Most respondents (57.6%) were female and most (54.2%) obtained information from healthcare facilities. Respondents demonstrated a moderate level of diabetes knowledge, with an overall median score of 62.5% across all question categories. A total of 41.3% respondents had a high level of knowledge, 29.1% had moderate knowledge levels and 29.6% had low knowledge levels. Using chi-square tests of association, tertiary-level education, younger age (between 18 and 29 years) and being employed were significantly associated with high knowledge levels.

**Conclusion:**

Health facilities in Mhlontlo should focus on providing health education for people aged more than 50 years to improve diabetes self-management.

**Contribution:**

This study contributes to previously unavailable context-specific information on diabetes knowledge levels among patients in Mhlontlo.

## Introduction

In 2022, 828 million people were living with diabetes worldwide, representing an increase of 638 million from 1990.^1^ Deaths from Type 2 diabetes also increased by 10.9% between 1990 and 2019.^2^ In South Africa, it is reported that the prevalence of diabetes increased from 3.86 to 4.46% between 2003 and 2016,^3^ and by 2020, the pooled prevalence of Type 2 diabetes in South Africa was estimated at 9.59%.^4^ This increase in diabetes prevalence is also coupled with an increase in diabetes-related mortality, with deaths attributed to diabetes increasing by 36.5% in South Africa between 2008 and 2018.^5^ In South Africa, the Eastern Cape province is included as one of the five provinces recording the highest number of deaths from non-communicable diseases^5^ such as diabetes. Several factors are associated with poor glycaemic control among people with diabetes, and these include a lack of knowledge about diabetes,^6,7,8,9^ the presence of co-morbidities^10^ and the lifestyle-related issues, such as alcohol use.^11^ Concerning the lack of knowledge, a previous study conducted in the Eastern Cape province, South Africa, found that knowledge levels among people with diabetes in the Buffalo Metropolitan City and Alfred Nzo were suboptimal, with an average score of 7.5 from a possible 20.^12^ Given the high prevalence of diabetes in South Africa, the associated increase in mortality and recognition that lack of knowledge contributes to poor glycaemic control, this study, conducted in the Mhlontlo local municipality, sought to describe the diabetes knowledge levels among people with Type 2 diabetes.

In South Africa, a study conducted in an urban setting in the Eastern Cape province found that receiving care at a primary health facility was associated with higher levels of diabetes knowledge compared to receiving care at a community health centre.^12^ Similar studies in other sub-Saharan African countries have also been conducted, with an urban Benin study revealing that 53% of people with diabetes had good knowledge.^13^ Alaofè et al.^13^ found that good knowledge of Type 2 diabetes was associated with being female, being married and having a longer duration of Type 2 diabetes. In urban Democratic Republic of Congo, as in South Africa,^12^ Ntontolo et al.^14^ found that people with Type 2 diabetes had low knowledge levels, with a mean score of 3.2 out of a possible 10. Moreover, higher knowledge levels of Type 2 diabetes were associated with higher levels of education, male gender and longer duration post diagnosis, while age above 70 years was associated with lower knowledge levels. A notable issue is that patient knowledge of diabetes differed across the different contexts, with the above studies noting how age, gender and education levels contributed to differences in diabetes knowledge levels.^12,13,14^

Although several studies^6,7,8,9^ describe the lack of diabetes knowledge as one of the contributing factors to poor glycaemic control, its management broadly includes medical management and self-management.^15^ Medical management of diabetes includes the use of pharmacotherapy,^15^ while self-management includes the use of behavioural and lifestyle measures to improve disease outcomes.^15^ For adequate self-management, the Society of Endocrinology, Metabolism and Diabetes of South Africa^16^ recommends Diabetes Self-management Education (DSME), which entails the provision of health education to enable patients to manage diabetes in the home environment.^16^ This health education may include education on nutrition, physical activity, medication and identification of complications.^16^ Notably, Webb et al.^16^ argue that DSME is one of the strongest predictors of diabetes disease progression and development of complications. Therefore, it is necessary to assess the knowledge levels of diabetes among patients to enable the identification of knowledge gaps, thus fostering adequate self-management practices to attain glycaemic control.

Previous studies have also assessed diabetes knowledge levels in various contexts, with some South African studies^12,15^ focusing on urban contexts^15^ while another Eastern Cape study,^12^ was conducted in the Buffalo City Metropolitan Municipality and Alfred Nzo District. From the researchers’ perspectives, there is a paucity of literature available from the OR Tambo district in the Eastern Cape. As such, considering the importance of diabetes education in ensuring self-management for glycaemic control, this study sought to describe diabetes knowledge levels among patients in the Mhlontlo local municipality, in the OR Tambo district in Eastern Cape, South Africa.

## Research methods and design

The study applied a quantitative approach utilising a cross-sectional design to collect data. This was guided by a positivist view of attaining objective data, providing a baseline for knowledge levels on diabetes in Mhlontlo municipality.

This study was conducted at Qumbu Health Centre and the Dr Malizo Mpehle Memorial Hospital Outpatients Department (OPD) in Tsolo. Both are public healthcare centres located in the Mhlontlo Local Municipality under the OR Tambo district municipality and provide outpatient services for people with diabetes.

Mhlontlo local municipality had a population of 186 391 in 2022, with 33.3% aged <15 years, 58.6% aged 15–64 and those over 65 made up 8.2% of this figure. It had a sex ratio of 90.5 males per 100 females.^17^ Among persons aged 20 and older, 11.4% had no schooling, 5.4% had higher education, and data for matric in 2022 were not available.^17^ According to the National Department of Health, there are 25 primary health care clinics and 2 community health centres in the Mhlontlo local municipality.^18^

The study applied a convenience sampling approach. This is defined as a sample that the researcher can practically access within a given period.^19^ This method was applied as a second choice, given the challenge of establishing a population and sample frame from which to collect data randomly, as the people with diabetes were available for routine clinic review in schedules of 3–6 months. Data were therefore collected from respondents whom the researchers could reach out to at the two facilities. The eligibility criteria were: (1) being physically available at one of the two sites during the data collection days; (2) having been diagnosed with Type 2 diabetes; and (3) being above the age of 18 years and having the ability to fully understand one’s rights as a research respondent.

Data were collected outside the reception sites of the two healthcare facilities. Patients in queues were physically approached by the researchers who identified themselves, explained their purpose and requested participation. Interested respondents went on to read or have their ethical rights explained, and upon agreeing and signing on these, they were given an English and IsiXhosa-structured questionnaire and a pen to complete.

Data were collected using the Diabetes Knowledge Questionnaire (DKQ-24), developed by Garcia et al.^20^ The DKQ-24 is a widely used instrument that has 24 questions and has been previously validated in the Greek context by Chrysi et al.,^21^ and some studies^22,23^ have also used the instrument. Knowledge questions have responses including ‘Yes’, ‘No’ and ‘I don’t know’. From the 24 questions on the DKQ-24, a ‘yes’ response is correct for 11 questions, while a ‘no’ response is correct for 13 questions.^20^

Data were analysed on the IBM Statistical Package for Social Sciences (SPSS) version 29. Descriptive statistics captured the percentages of knowledge, the lack of, or doubt about it, per statement. At the data analysis stage, the knowledge questions were separated into three categories: (1) causes, risk factors and general diabetes knowledge, (2) signs and symptoms and (3) diabetes self-management and a mean score and standard deviation were computed for each. An overall score was also computed for all the questions. Chi-square tests of association were computed to test the association between sociodemographic characteristics on the one hand and, on the other hand, knowledge levels at a 0.05 significance level. To define knowledge levels, we categorised low level as 0% – 49%, moderate as 50% – 74% and high as 75% – 100%.

To enhance validity, the researchers conducted a pilot study to assess whether the questionnaire measured what it is purported to measure. The researchers assessed respondents’ ability to understand the instructions given to them as well as the questions and statements on the questionnaire. The researcher also time-tested the questionnaire with the intention of ensuring it took on average 12 min to complete. The study also relied on previous studies on diabetes knowledge assessments to ascertain the quality of questioning as well as the types of questions to present.

To enhance the reliability, the researchers ensured clear and consistent wording of questions, conducted the above-mentioned pilot tests and maintained a standardised administration process defined under the data collection process above.

### Ethical considerations

Ethical clearance to conduct this study was obtained from the Walter Sisulu University Faculty of Health Sciences Human Research Ethics and Biosafety Committee. The ethical clearance number is 061/2024. The participants gave written consent to participate in the study.

## Results

### Sample demographic characteristics

A total of 172 respondents comprised the sample. Out of 172 respondents, 57.6% (*n* = 99) were female. In terms of age, the most represented age group was 50 years and above (46.5%, *n* = 80). Almost half of the respondents, or 45.9% (*n* = 79), had completed secondary education, while 25.6% (*n* = 44) had attained tertiary education. All respondents had Type 2 diabetes. Table 1 shows the sample characteristics.

**TABLE 1:** Demographics variable (*N* = 172).

Variable	*n*	%
**Gender**		
Male	73	42.2
Female	99	57.6
**Age (years)**		
18–29	11	6.4
30–39	44	25.6
40–49	37	21.5
≥ 50	80	46.5
**Level of education**		
No formal education	11	6.4
Primary education and lower	38	22.1
Secondary education	79	45.9
Tertiary education	44	25.6
**Employment status**		
Employed	78	45.3
Unemployed	81	47.1
Self-employed	13	7.6
**Facility**		
Facility 1	107	62.2
Facility 2	65	37.8

### Respondents’ sources of knowledge

Out of 172 respondents, 9.3% selected mainstream media as their main source of diabetes information. Others – 10.5% selected the Internet, including Apps, 16.3% – social and support groups – family included, 9.9% current and past educational institutions and 54.2% healthcare facilities. Thus, most respondents’ main source of diabetes knowledge was a healthcare facility. Table 2 shows the respondents’ sources of knowledge.

**TABLE 2:** Respondents’ sources of knowledge.

Main knowledge source	Frequency	%
Mainstream media	16	9.3
Internet and Apps	18	10.5
Social and support groups	28	16.3
Educational institution	17	9.9
Healthcare facility	93	54.0
**Totals**	**172**	**100.0**

### Knowledge about diabetes signs and symptoms

Out of 172 respondents, 66.2% correctly responded that frequent urination and thirst are not signs of low blood sugar; 66.9% correctly identified that shaking and sweating are not signs of high blood sugar, while 61.0% were aware that cuts and abrasions heal slowly. Also, 62.2% affirmed that diabetes can cause loss of feeling in the extremities, and 60.5% knew that diabetes can damage the kidneys. Respondents also correctly observed that diabetes causes poor circulation. Overall, the mean percentage of correct responses was 61.7% (standard deviation [s.d.] = ± 4.8). Generally, most patients answered correctly, although 23.3% gave an incorrect response, and another 23.3% did not know the answers. Table 3 shows respondents’ responses to statements testing their knowledge on the signs and symptoms of diabetes.

**TABLE 3:** Knowledge about diabetes signs and symptoms (*N* = 172).

Test statement	Correct response (%)	Incorrect response (%)	I don’t know (%)	Total
Frequent urination/thirst are signs of low blood sugar	66.2	19.8	14.0	100.0
Shaking and sweating are signs of low blood sugar	66.9	16.2	16.9	100.0
Cuts and abrasions on diabetics heal more slowly	61.0	18.6	20.4	100.0
Diabetes can cause loss of feeling in the extremities	62.2	18.6	19.2	100.0
Diabetes can damage the kidneys	60.5	20.3	19.2	100.0
Diabetes often causes poor circulation	53.5	23.3	23.2	100.0
Mean	61.7	19.5	18.8	-
Standard deviation	4.8	2.1	2.9	-

### Diabetes self-management knowledge

Among the 172 respondents, 64.5% correctly responded that a diabetic diet does not consist mostly of special foods, while 61.6% correctly identified that regular exercise does not reduce the need for diabetic medication, and 63.4% affirmed that blood sugar usually increases in untreated diabetes. As observed, 59.3% responded correctly with a ‘no’ that tight socks or hose are not bad for diabetics, and 54.7% acknowledged that food preparation is as important as food choice. Additionally, 64.0% of the respondents correctly noted ‘no’ to the statement that medication is more important than diet and exercise to control my diabetes. Most (53.5%) respondents also highlighted that diabetics should take extra care when cutting their toenails, while another 53.5% were correct that a person with diabetes should not cleanse cuts with iodine and alcohol. A total of 51.2% also indicated correctly that it is not the best way to check diabetes by checking urine. On average, 58.4% (s.d. = ± 4.9) of respondents answered the statements on diabetes self-management knowledge correctly. Table 4 shows the participants’ responses to statements testing their knowledge on diabetes self-management.

**TABLE 4:** Diabetes self-management knowledge.

Test statement	*N*	Correct (%)	Incorrect (%)	Don’t know (%)	Total
In untreated diabetes, blood sugar usually increases	172	63.4	20.9	15.7	100.0
Regular exercise reduces the need for insulin and other diabetic medications	172	61.6	18.0	20.4	100.0
The way I prepare my food is as important as the food I eat	172	54.7	28.5	16.8	100.0
Tight elastic socks or hose are not bad for diabetics	172	59.3	21.5	19.2	100.0
A diabetic diet consists mostly of special foods	172	64.5	21.0	14.5	100.0
Medication is more important than diet and exercise to control my diabetes	172	64.0	24.4	11.6	100.0
Diabetics should take extra care when cutting their toenails	172	53.5	30.2	16.3	100.0
A person with diabetes should cleanse cuts with iodine and alcohol	172	53.5	28.5	18.0	100.0
The best way to check my diabetes is to check my urine	172	51.2	32.2	16.6	100.0
Mean	-	58.4	25.0	16.9	-
Standard deviation	-	4.9	4.7	2.4	-

### Knowledge on causes, risks and general diabetes knowledge

Of the 172 respondents, 55.8% correctly indicated that eating too much sugar and other sweet foods is not a cause of diabetes. A total of 58.2% of the respondents also correctly found that the usual cause of diabetes is a lack of effective insulin in the body, while another 58.1% indicated that diabetes cannot be caused by the failure of the kidneys to keep sugar out of the urine. Concerning the likelihood of children born to diabetic people being diabetic, 62.2% of respondents gave a correct response. A total of 59.8% and 67.4% respondents, respectively, also correctly observed that kidneys do not produce insulin and that diabetes cannot be cured. In addition, 68% of the respondents correctly observed that there were 2 types of diabetes (Type 1 and Type 2). Respondents performed poorly in two questions, where most (46.7%) incorrectly stated that a fasting blood sugar of 210 is not too high, and 48.3% correctly noted that an insulin reaction is caused by too much food. The overall mean score for correct responses on diabetes general knowledge, causes and risks was 56.1% (s.d. = ± 11.8). Table 5 shows respondents’ responses to statements testing their general knowledge on diabetes as well as their knowledge on the causes and risks of diabetes.

**TABLE 5:** Knowledge on causes, risks and general diabetes knowledge.

Test statement	*N*	Correct responses (%)	Incorrect responses (%)	I don’t know (%)	Total
Eating too much sugar and other sweet foods is a cause of diabetes	172	55.8	31.4	12.8	100.0
The usual cause of diabetes is a lack of effective insulin in the body	172	58.2	27.3	14.5	100.0
Diabetes can be caused by the failure of the kidneys to keep sugar out of the urine	172	58.1	26.2	15.7	100.0
If I’m diabetic, my children have a higher chance of being diabetic	172	62.2	24.4	13.4	100.0
Kidneys produce insulin	172	59.8	26.2	14.0	100.0
Diabetes can be cured	172	67.4	23.3	9.3	100.0
A fasting blood sugar of 210 is too high	172	26.7	46.7	26.6	100.0
There are two main types of diabetes: Type 1 and Type 2	172	68.0	15.7	16.3	100.0
An insulin reaction is caused by too much food	172	48.2	32.0	19.8	100.0
Mean	-	56.1	28.1	15.8	-
Standard deviation	-	11.8	7.9	4.7	-

### Levels of diabetes knowledge

Overall, for the three categories of questions, the median score of correct responses was 62.5%. Most (41.3%, *n* = 71) respondents had a high level of knowledge, while 29.1% (*n* = 50) had moderate levels of knowledge and 29.6% (*n* = 51) had low levels of knowledge. Table 6 shows the overall diabetes knowledge levels among respondents.

**TABLE 6:** Overall diabetes knowledge score.

Level of knowledge	*n*	%
High	71	41.3
Moderate	50	29.1
Low	51	29.6
**Total**	**172**	**100.0**

### Chi-square test – Knowledge levels versus demographic factors

Statistically significant associations were found between level of knowledge and age (*p* < 0.001), level of knowledge and level of education (*p* < 0.001) and level of knowledge and employment status (*p* < 0.001). The strengths of these associations were strongest between the level of knowledge and the level of education (Cramer’s *V* = 0.754). People with a tertiary education tended to have a high level of diabetes knowledge, while people with no formal education had low levels of knowledge. The strength of association between age and level of knowledge (Cramer’s *V* = 0.471) was also strong. In this regard, people aged 18–29 years had higher knowledge levels, while persons aged more than 50 years had lower knowledge levels. Concerning employment status, people who were employed had higher levels of knowledge, while unemployed people had a low level of knowledge. Table 7 summarises chi-square test results for the association between gender, age, level of education, facility, employment status and main source of diabetes information versus patients’ knowledge levels.

**TABLE 7:** Chi-Square test – Knowledge levels versus demographic factors.

Demographic variable	Chi-Square	*Df*	Sig.	Cramer’s *V*	Sig.
Gender	4.25	2	0.119	0.16	0.119
Age*	76.40	6	< 0.001*	0.47	< 0.001*
Level of education*	195.73	6	< 0.001*	0.75	< 0.001*
Main source of diabetes information	12.76	8	0.12	0.19	0.12
Facility	2.39	2	0.302	0.12	0.302
Employment status*	32.32	4	< 0.001*	0.31	< 0.001*

* , statistically significant (*p* < 0.05).

## Discussion

This study sought to describe the level of knowledge among people living with diabetes in Mhlontlo, Eastern Cape province, South Africa. A sample of 172 respondents was included in the study; most respondents were female, with 25.6% of respondents having a tertiary qualification. In the South African general population, at least 12.7% of the population have attained a tertiary qualification.^24^ Therefore, the inclusion of a higher population with tertiary education limits the applicability of our findings, as it may not reflect the education level in South Africa. The study found a mean score of 61.7% (s.d. = ± 4.8) on diabetes signs and symptoms, 58.4% (s.d. = ± 4.9) on knowledge of self-management and 56.1% (s.d. = ± 11.8) on knowledge of causes, risks and general diabetes knowledge. Overall, the median score was 62.5%. There were statistically significant associations between younger age (18–29 years old), being employed and having a tertiary education and high knowledge levels.

The findings from this study show that respondents demonstrated a moderate level of knowledge about the signs and symptoms of diabetes, with a mean correct response of 61.7% (s.d. = ± 4.8). When compared to the literature, these results suggest a knowledge gap, specifically when compared to studies by Alenbalu et al.,^25^ where knowledge levels were very high, with over 80% of respondents correctly identifying major diabetes symptoms such as excessive urination (81.8%), thirst (82.9%) and vision problems (86.8%). Similarly, Alaofe et al.^13^ reported high levels of knowledge about complications, such as kidney failure (92%) and heart failure (46%), emphasising a higher knowledge level in their sample. On the other hand, the results from Ntontolo et al.^14^ reported a lower knowledge level of 34.9% regarding signs and symptoms, suggesting that knowledge levels could vary significantly by context. Overall, participants in this study could improve their knowledge on Diabetes signs and symptoms.

When compared with the existing literature, the findings reflect considerably better but still limited knowledge on some factors. For example, Ntontolo et al.^14^ reported an even lower level of knowledge around causes and risk factors, supporting the view that this remains a key gap in populations. In contrast, Alaofe et al.^13^ found that only 7.7% of respondents correctly identified low insulin production as a cause, significantly lower than the 58.2% in this study, suggesting better knowledge among this sample. However, while knowledge of hyperglycaemia (91%) and the incurability of diabetes (93%) was high in Alaofe’s^13^ study, this was not the case in this study, where knowledge was low and moderate, respectively, rather than high.

Findings from a study by Alenbalu et al.,^25^ suggest that although 76% of respondents knew that poor management leads to complications, only 62% recognised cardiovascular risks, close to the 62.2% in this study who acknowledged hereditary risk. In addition, Roux’s^26^ study in the Free State found that only 37.6% understood the family link, while Benin respondents showed 61% knowledge on this issue,^13^ aligning closely with current results. Overall, while respondents in this study demonstrate a basic knowledge of major causes and risk factors, concerning knowledge gaps remain.

The study results suggest a moderate level of self-management knowledge among respondents, with a mean correct response rate of 58.4% (s.d. = ± 4.9). This performance is higher than the 42.2% general diabetes management knowledge reported by Ntontolo et al.,^14^ indicating that the sample’s knowledge on diabetes management could be better than that held in other contexts. However, when compared to studies such as Alenbalu et al.,^25^ where over 90% of respondents understood the importance of avoiding harmful substances, maintaining regular exercise and lifelong medication use, the knowledge levels in the current study appear less comprehensive. Similarly, while Mphasha et al.^27^ found that over 80% of respondents understood the significance of dietary choices, only 54.7% in this study recognised that food preparation is as important as food choice. These findings suggest that although most sampled respondents held the correct knowledge, there was still room for improvement to reach the knowledge levels achieved in other contexts.

The study shows that age and level of education influence diabetes knowledge levels. Age more than 50 years was significantly linked to low knowledge levels (*p* < 0.001), suggesting older individuals may not have adequate health information. Education level was significantly associated with higher levels of diabetes knowledge. These results align with Liu et al.^28^ and Poulimeneas et al.,^29^ who found that higher education improves diabetes knowledge. In addition, our study found that patients who were employed had a statistically significant higher level of knowledge than those who were unemployed or self-employed. This aligns with findings from previous study by Sharma et al.^30^ in India, who also found higher levels of knowledge among employed people with a tertiary qualification.

The overall median knowledge score of 62.5% in this study indicates a moderate understanding of diabetes among respondents, which is slightly higher than findings from related studies. For instance, Alhaik et al.^31^ reported a self-care knowledge rate of 58.28% in Jordan, while Alaofe et al.^13^ found an overall 53% knowledge level in Southern Benin. Similarly, Maduemezia et al.^32^ recorded a 57% overall knowledge score at a Johannesburg hospital. These suggest that although awareness exists among most patients sampled, knowledge gaps, especially around symptoms and management, persist. In contrast, Moshoeshoe et al.^15^ observed poorer knowledge levels at another Johannesburg hospital, where only 2.34% of respondents had good knowledge and 37.38% had poor understanding. Likewise, Ntontolo et al.^14^ reported very low knowledge rates in the Democratic Republic of Congo, linking this to age, gender and education. Compared to these, this study highlights slightly better but still incomplete knowledge.

### Limitations

The study relied on a non-random, convenience sampling technique and respondents’ self-reported data. Also, while the study compared the level of patient knowledge with other research from the literature, it should be noted that each of the studies applied a different set of questions to test such knowledge. This lack of standardised reporting affects comparisons made as part of the study’s discussion. Moreover, the study was limited by a sample with a high proportion of people with a tertiary qualification, which does not reflect the percentage of people with a tertiary education in the South African general population.

## Conclusion

The study reveals that while respondents demonstrated a moderate overall knowledge of diabetes, notable gaps persisted in understanding symptoms, causes and self-management practices. Given that patients’ main source of information is health facilities, this study reinforces how healthcare facilities play a critical role in patients’ diabetes knowledge across health seekers of different educational attainment, ages and genders. In this regard, given the recent funding cuts in the public health sector in South Africa, innovative ways to improve patients’ level of diabetes knowledge ought to be used to ensure patients attain optimal levels of knowledge from the health facilities. This may include the use of digital health technologies such as videos, which may be sent to patients to provide customised health education.
